# Hypothalamic-pituitary-adrenal axis and renin-angiotensin-aldosterone system in adulthood PTSD and childhood maltreatment history

**DOI:** 10.3389/fpsyt.2022.967779

**Published:** 2023-01-09

**Authors:** Ryoko Kakehi, Hiroaki Hori, Fuyuko Yoshida, Mariko Itoh, Mingming Lin, Madoka Niwa, Megumi Narita, Keiko Ino, Risa Imai, Daimei Sasayama, Toshiko Kamo, Hiroshi Kunugi, Yoshiharu Kim

**Affiliations:** ^1^Department of Behavioral Medicine, National Center of Neurology and Psychiatry, National Institute of Mental Health, Tokyo, Japan; ^2^Department of Medical Science, Tohoku University Graduate School of Medicine, Sendai, Japan; ^3^Department of Nursing, Wayō Women’s University, Chiba, Japan; ^4^Department of Mental Disorder Research, National Center of Neurology and Psychiatry, National Institute of Neuroscience, Tokyo, Japan; ^5^Center for Environmental and Health Sciences, Hokkaido University, Sapporo, Japan; ^6^Department of Psychiatry and Cognitive-Behavioral Medicine, Nagoya City University Graduate School of Medical Sciences, Nagoya, Japan; ^7^Risa Irinaka Mental Clinic, Nagoya, Japan; ^8^Department of Psychiatry, Shinshu University School of Medicine, Nagano, Japan; ^9^Wakamatsu-cho Mental and Skin Clinic, Tokyo, Japan; ^10^Department of Psychiatry, Teikyo University School of Medicine, Tokyo, Japan

**Keywords:** posttraumatic stress disorder (PTSD), childhood maltreatment, hypothalamic-pituitary-adrenal (HPA) axis, renin-angiotensin-aldosterone (RAA) system, dehydroepiandrosterone-sulphate (DHEA-S), gene, polymorphism

## Abstract

Accumulated evidence shows that psychological trauma and posttraumatic stress disorder (PTSD) are associated with dysfunction in the hypothalamic-pituitary-adrenal (HPA) axis. Besides the HPA axis hormones, recent evidence suggests that the renin-angiotensin-aldosterone (RAA) system and genetic factors may be involved in trauma/PTSD as well as in HPA axis regulation. This study attempted to better understand the HPA axis function in relation to PTSD and childhood maltreatment by simultaneously examining RAA system and genetic polymorphisms of candidate genes. Here we studied 69 civilian women with PTSD and 107 healthy control women without DSM-IV-based traumatic experience. Childhood maltreatment history was assessed with the Childhood Trauma Questionnaire. PTSD severity was assessed with the Posttraumatic Diagnostic Scale. Functional disability was assessed with the Sheehan Disability Scale. HPA axis was examined by measuring blood levels of cortisol, adrenocorticotropic hormone, and dehydroepiandrosterone-sulphate (DHEA-S). RAA system was examined by measuring blood renin and aldosterone levels. The *FKBP5* rs1360780 and *CACNA1C* rs1006737 polymorphisms were genotyped. No significant differences were seen between patients and controls in any of the five hormone levels. DHEA-S levels were significantly negatively correlated with overall PTSD severity (*p* = 0.003) and functional disability (*p* = 0.008). A two-way analysis of variance with diagnostic groups and genotypes as fixed factors revealed that patients with the rs1006737 A-allele had significantly lower DHEA-S levels than patients with the GG genotype (*p* = 0.002) and controls with the A-allele (*p* = 0.006). Childhood maltreatment history was not significantly correlated with any of the five hormone levels. These results were generally unchanged after controlling for the potentially confounding effect of age, depression, and anxiety. Our findings suggest that lower DHEA-S levels could indicate more severe subtype of PTSD, the association of which might be partly modified by the *CACNA1C* polymorphism.

## 1. Introduction

Posttraumatic stress disorder (PTSD) is a serious psychiatric condition that can develop after a major traumatic event, often leading to a chronic course and severe functional impairment. While the etiology of PTSD remains unclear, this disorder likely involves alterations in stress response systems since it is associated with severe traumatic stress.

Among these systems, the hypothalamic-pituitary-adrenal (HPA) axis is crucially involved in the coordination of stress responses and the maintenance of homeostasis. The HPA axis activation results in an increased secretion of glucocorticoid (cortisol in humans) from the adrenal cortex, and glucocorticoid in turn regulates its own production through negative feedback by binding to glucocorticoid receptors (GRs) in the pituitary, hypothalamus, and hippocampus as well as mineralocorticoid receptors in the hippocampus. HPA axis dysfunction, including lower cortisol levels and greater GR sensitivity, is a well-known biological feature of PTSD (1, 2). However, findings to date have been controversial especially with respect to basal cortisol levels, such that lower levels in individuals with PTSD compared to controls have been shown in some (3, 4) but not all (5) meta-analyses. This inconsistency might be attributable to several methodological issues, such as possible effects of trauma exposure itself in control individuals and different paradigms of cortisol measurement (6).

In addition to cortisol, dehydroepiandrosterone (DHEA) and its more stable sulphated conjugated metabolite, DHEA-S [herein together referred to as “DHEA(S)”], have been implicated in the pathophysiology of PTSD (6, 7). Like cortisol, DHEA(S) is secreted by the adrenal cortex, at least partly in response to adrenocorticotropic hormone (ACTH). DHEA(S) is the most abundant adrenal steroid in humans. Compared to DHEA, DHEA-S shows much higher concentrations in serum, has longer half-life, and exhibits smaller diurnal variation. DHEA(S) has neuroprotective actions, and is also classified as a neurosteroid, in that it not only crosses the blood brain barrier to exert effects within the central nervous system but is synthesized *de novo* in the brain from its sterol precursor (8). DHEA(S) acts as a gamma-aminobutyric acid type A receptor antagonist and is involved in the regulation of neuronal survival and differentiation (9). It is also shown to act as a sigma-1 receptor agonist (10). Furthermore, DHEA(S) is thought to possess anti-glucocorticoid properties, as it antagonizes the effect of cortisol (11), inhibits glucocorticoid-induced enzyme activity (12), and rapidly reduces cortisol levels when acutely administered (13). Thus, DHEA(S) is assumed to be involved in stress responses (14) and, as such, investigated in various psychiatric disorders including PTSD (6, 7). However, the findings on DHEA(S) levels in PTSD patients relative to controls are mixed, with higher (15, 16), lower (17), and similar (18, 19) levels have all been reported.

Besides the HPA axis, the renin-angiotensin-aldosterone (RAA) system is activated in response to stress, resulting in increased levels of angiotensin II and aldosterone (20). The RAA system is an endocrine system centrally involved in the regulation of systemic blood pressure and blood fluid volume. This system has a bidirectional interaction with the HPA axis, such that angiotensin activates the HPA axis to increase circulating glucocorticoids (20) and that ACTH stimulates aldosterone production (21). Recently, several lines of research have suggested that the RAA system can be associated with PTSD. In clinical studies, the use of angiotensin-converting enzyme inhibitors and angiotensin receptor blockers (ARBs) was found to be associated with significantly decreased PTSD symptoms (22, 23). In a neuroimaging study targeting highly anxious individuals, an ARB losartan affected emotional processing and amygdala activity (24). In rodent models, the RAA system has been shown to be involved in the regulation of fear memory (25, 26). Furthermore, biomarker studies have reported that circulating renin levels are increased in PTSD patients (27) and that aldosterone levels are increased in relation to childhood trauma (28, 29). In contrast to these findings, however, a recent longitudinal study reported that women with chronic PTSD had significantly lower plasma aldosterone levels averaged over time, compared to women unexposed to trauma (30). As there have been only a few studies investigating RAA system hormones in PTSD, more needs to be done to clarify the nature of trauma-related alterations in this system.

The HPA axis is affected by multiple genes as well as environmental factors; for example, basal cortisol is reported to have a heritability of around 60% by twin studies (31). Of these genetic factors, the *FKBP5* (FK506 binding protein five) gene, whose protein acts as a co-chaperone of GR complex, plays a pivotal role in the regulation of HPA axis. The *FKBP5* overexpression can decrease glucocorticoid binding affinity and nuclear translocation of GRs, thereby reducing GR sensitivity and negative feedback of HPA axis (32). *FKBP5* expression is moderated by common genetic variants that are part of a haplotype including rs1360780, a single nucleotide polymorphism (SNP) considered as a functional variant of this gene (33). Studies have investigated the effect of rs1360780 on stress-related psychiatric disorders, demonstrating that it can interact with childhood trauma and be associated with PTSD (32, 33). Another gene that could be involved in both PTSD (34) and HPA axis (35) is the *CACNA1C* (calcium voltage-gated channel subunit alpha1C) gene. This gene encodes an alpha-1 subunit of the voltage-gated L-type calcium channel (Cav1.2), which mediates the influx of calcium ions into the cell upon membrane polarization and plays a role in dendritic development, neuronal survival, synaptic plasticity, and memory/learning (36). An intronic SNP rs1006737 of *CACNA1C* has been identified as a risk factor for multiple psychiatric disorders and their various phenotypes in many studies including our previous one (37–39). The G>A substitution of rs1006737 is shown to be associated with decreased expression of *CACNA1C* (40, 41). Importantly, a recent study showed that mice with global heterozygous loss of *Cacna1c* and mice with specific deletion of *Cacna1c* from dopamine D1-receptor-expressing neurons both exhibited exaggerated remote contextual fear, which corresponds to the re-experiencing symptom of PTSD (34). These findings together raise the possibility that the inter-individual differences in HPA axis in PTSD may be explained in part by the functional SNPs of *FKBP5* and *CACNA1C*.

The present study aimed to examine HPA axis function by measuring blood levels of cortisol, ACTH, and DHEA-S, in PTSD and childhood maltreatment history. We also investigated RAA system by measuring blood renin and aldosterone levels. These hormones were also examined in relation to PTSD severity and functional disability. We further explored the effects of genetic variations in *FKBP5* (rs1360780) and *CACNA1C* (rs1006737) on HPA axis hormones and PTSD symptomatology. Given the modest sample size, we restricted the genetic investigation to these two functional loci implicated in both HPA axis and PTSD. Another issue that needs to be considered is the paradigm of HPA axis hormone measurement. Although dysregulated negative feedback is shown to reflect an important aspect of HPA axis abnormalities in trauma/PTSD (42, 43), we only measured baseline levels of these hormones in the main sample because it was difficult to conduct laborious challenge tests in the clinical sample. Instead, prior to the main study, we conducted a dexamethasone (DEX) suppression test in a small independent sample of healthy individuals. This test provided information regarding to what extent the basal levels of HPA axis hormones would predict their levels after DEX challenge. While DEX-induced negative feedback of cortisol is well documented, this is not the case with DHEA-S, and therefore we attempted to know the physiological response of DHEA-S to the DEX challenge in a general population.

## 2. Materials and methods

### 2.1. Participants

The present study was conducted at three institutes: National Center of Neurology and Psychiatry, Tokyo Women’s Medical University, and Nagoya City University. This study was approved by the ethics committees of these institutes, and was conducted in accordance with the Declaration of Helsinki. Written informed consent was obtained from all participants after they had received a detailed explanation of the study. The study procedures were identical across the three sites.

This study was conducted as part of an ongoing larger project. Data collection for the present study was conducted between 2015 and 2020. This study included only women for both patients and controls; the sole inclusion of women, or exclusion of men, was because the vast majority of the sample included in our entire project were women.

A total of 69 civilian women with PTSD (age range: 18–56 years) participated in this study. Of them, 64 were outpatients at these hospitals and clinics, and their attending doctors were asked to inform the researcher of all potentially eligible patients. The remaining five patients were outpatients at the nearby clinics and were recruited through advertisements on our website. All patients had already been diagnosed as having PTSD by attending clinicians. The experience of traumatic events and diagnosis of PTSD were confirmed by the validated Japanese version (44) of the Posttraumatic Diagnostic Scale (PDS) (45). In addition, the validated Japanese version (46) of the Mini International Neuropsychiatric Interview (M.I.N.I) (47) was administered by an expert clinician to identify any other Axis-I disorders as well as PTSD. Patients with comorbid schizophrenia and those with marked manic episodes of bipolar disorder were excluded from the study.

In addition, 107 healthy control women without PDS-based traumatic experience (20–59 years) were recruited mostly through advertisements in free local magazines and on our website, and a few of them were recruited by word of mouth. The PDS was administered to healthy controls in order to examine the presence/absence of traumatic experiences and, if present, they were excluded from this study. All healthy individuals were interviewed by a board-certified psychiatrist which included M.I.N.I and non-structured interview, and those who demonstrated current Axis-I disorders or apparent signs of past psychiatric disorders were excluded.

### 2.2. Psychological assessment

Psychological and clinical characteristics of the patients and controls were assessed using the following five self-report questionnaires:

#### 2.2.1. Posttraumatic Diagnostic Scale (PDS) (45).

The PDS comprises 4 parts that evaluate traumatic experiences reflecting Criteria A of the DSM-IV (Parts 1 and 2), PTSD severity reflecting Criteria B-D (Part 3), and functional impairments associated with PTSD symptoms (Part 4). Part 3 comprises 17 items pertaining to PTSD symptomatology during the past month, and each item is scored on a 4-point scale of frequency from 0 to 3, with higher scores indicating more frequently occurring (or more severe) symptoms.

In the present study, we administered Parts 1 and 2 to all participants in order to determine the presence/absence of traumatic experiences, and if present, Parts 3 and 4 were administered for the assessment of diagnosis and severity of PTSD. We used the Japanese version of PDS that has demonstrated good reliability and validity (44, 48).

#### 2.2.2. Childhood Trauma Questionnaire (CTQ) (49).

Childhood maltreatment history was assessed with our Japanese version (CTQ-JNIMH) (50) of the CTQ (49), a widely used questionnaire for assessing history of childhood maltreatment. The CTQ has demonstrated adequate psychometric properties as demonstrated by a good fit of the 5-factor structure (49, 51), internal consistency (49, 52), and test-retest reliability (53). The commonly used 28-item version of CTQ includes 25 clinical items and 3 validity items. The 25 items load onto five subscales that assess different types of childhood maltreatment, including emotional abuse, physical abuse, sexual abuse, emotional neglect, and physical neglect. All items start with the phrase “when I was growing u,p” and are rated on a 5-point scale ranging from 1 to 5 (1 = “never true” to 5 = “very often true”), with higher scores indicating greater maltreatment. Accordingly, the minimum/maximum possible score of each CTQ subscale, i.e., emotional abuse, physical abuse, sexual abuse, emotional neglect, and physical neglect, is 5–25. Scores of these five subscales are summed to yield a total CTQ score.

Cronbach α coefficients of the 5 CTQ subscales, namely emotional abuse, physical abuse, sexual abuse, emotional neglect, and physical neglect, in the present sample (i.e., combined sample of patients and controls) were 0.93, 0.86, 0.93, 0.92, and 0.70, respectively. There was one participant (patient) who did not complete the sexual abuse subsection.

#### 2.2.3. Beck Depression Inventory-II (BDI-II) (54).

The BDI-II is a 21-item self-report questionnaire widely used to measure depression severity during the past 2 weeks (54). Each item is scored on a 4-point scale from 0 to 3, with higher scores indicating more severe depressive symptoms. We used the validated Japanese version (55) of BDI-II.

#### 2.2.4. State-Trait Anxiety Inventory (STAI) (56).

The STAI is a self-report questionnaire widely used to assess anxiety (56). It consists of two subscales for trait (STAI-T) and state (STAI-S) anxiety, both of which comprise 20 items that are scored on a 4-point scale from 1 to 4; higher scores indicate greater anxiety. We used the validated Japanese version (57) of STAI.

#### 2.2.5. Sheehan Disability Scale (SDISS) (58).

The SDISS is a discretized visual analog rating scale of functional disability in three domains, namely work/school, social, and family life (58). Each domain is rated from 0 (unimpaired) to 10 (extremely impaired), which are summed to yield a global disability score ranging from 0 to 30. In this study the validated Japanese version (59) of SDISS was administered to all participants. There were two participants (both patients) who did not complete this questionnaire.

### 2.3. Measurement of biomarkers

Blood sampling was performed on the same day as the psychological assessments. The samples were collected from each participant around noon before lunch, between 11:30 a.m. and 12:30 p.m., in order to minimize possible effects of the time of day and dietary intake.

Levels of cortisol, ACTH, DHEA-S, renin, and aldosterone were measured at a clinical laboratory (SRL Inc., Tokyo, Japan). Serum cortisol levels and plasma ACTH levels were measured by electro chemiluminescence immunoassay. Serum DHEA-S levels, plasma renin levels, and plasma aldosterone levels were measured by chemiluminescent enzyme immunoassay.

Measurements of cortisol, ACTH and DHEA-S were performed at the laboratory soon after the blood was collected from each participant, while measurements of renin and aldosterone were performed afterward at the same laboratory using extracted plasma samples that had been stored at −80°C in a deep freezer at our institute. Data of cortisol, ACTH, and DHEA-S were available for all participants, while those of renin and aldosterone were available for 61 patients (88.4%) and 96 controls (89.7%). This sample attrition was due to lack of residual plasma samples for a subset of participants.

The detection limit for cortisol was 0.06 μg/dl. There were no participants whose cortisol levels were below this detection limit. The intra- and inter-assay coefficients of variation for cortisol were both less than 2.7%. For ACTH, the detection limit was 1.5 pg/ml; only one participant showed an ACTH level below this limit. The intra- and inter-assay coefficients of variation for ACTH were both less than 2.5%. For DHEA-S, the detection limit was 2.0 μg/dl; one participant showed a DHEA-S level below this limit. The intra- and inter-assay coefficients of variation for DHEA-S were both less than 5.0%. For renin, the detection limit was 0.20 pg/ml; no participants showed renin levels below this limit. The intra- and inter-assay coefficients of variation for renin were both less than 3.2%. For aldosterone, the detection limit was 4.0 pg/ml; no participants showed aldosterone levels below this limit. The intra- and inter-assay coefficients of variation for aldosterone were both less than 2.8%. Values under the detection limits were treated as 0 (μg/dl or pg/ml).

### 2.4. Genotyping

A majority of the participants also participated in the genetic testing by blood sampling, and genotyping was performed for 51 patients and 94 controls. The reason for this sample attrition was that informed consent to the genetic study was not provided for a portion of participants. Genomic DNA was prepared from venous blood according to standard procedures. Rs1360780 of *FKBP5* (assay ID: C_8852038_10) and rs1006737 of *CACNA1C* (C___2584015_10) were genotyped using the TaqMan SNP Genotyping Assays. The thermal cycling conditions for polymerase chain reaction were: 1 cycle at 95°C for 10 min followed by 45 cycles of 95°C for 15 s and 60°C for 1 min. The allele-specific fluorescence was measured with ABI PRISM 7,900 Sequence Detection Systems (Applied Biosystems, Foster City, CA). These genotyping data have been deposited to the ClinVar database with accession numbers SCV002587063 (for rs1360780) and SCV002588462 (for rs1006737).

### 2.5. Additional experiments for HPA axis negative feedback in an independent sample

Prior to the main study, we conducted a DEX suppression test to measure cortisol and DHEA-S levels before and after DEX administration as a complementary examination of HPA axis, using an independent sample of 33 women (age range: 20–67; mean 44.8 ± 12.8 years) recruited *via* advertisements in free local magazines and on our website. This study was approved by the ethics committee of National Center of Neurology and Psychiatry, and written informed consent was obtained from all participants. They were confirmed to be psychiatrically healthy by the M.I.N.I; while information on the presence/absence of traumatic experiences was not available for these individuals, as the PDS was not administered to them.

The test procedure was as follows: on the first day, participants visited our laboratory and blood samples were collected at 10:00 h, and they took 0.5 mg tablet of DEX at 23:00 h at home; on the next day, blood samples were collected at 10:00 h at our laboratory again. High correlation between the basal and post-DEX levels indicates that the post-DEX level can be predicted by its basal level, irrespective of the extent of DEX-induced negative feedback; while low correlation suggests that the negative feedback is independent of the basal level. We used 0.5 mg of DEX, given that the DEX suppression test with this dose of DEX has been widely employed in the studies of stress-related psychiatric disorders including PTSD (42, 43). We collected blood samples at 10:00 h, considering that the previous studies of DEX suppression test in relation to trauma and PTSD have conducted blood sampling in the morning (42, 43).

### 2.6. Statistical analysis

Averages are reported as “means ± SD,” or “median (25th–75th percentile)” where appropriate. Categorical variables were compared using the χ^2^ test. Group differences were examined using the *t*-test, Mann–Whitney U-test, Kruskal–Wallis test, or analysis of variance (ANOVA), according to the nature and distribution of data. Correlations were calculated using Pearson’s r or Spearman’s rho.

Primary analyses were conducted as follows. We first tested the difference in blood biomarker levels between patients and controls using the Mann–Whitney *U*-test. Next, correlations of clinical/psychological characteristics with biomarkers were calculated in patients and in controls (data of PTSD severity was available only for patients), using the Spearman’s rho. Then, the correlation coefficient in patients was compared with that in controls in order to further examine whether the correlation is more apparent in patients than in controls. This analysis was done by converting Spearman’s correlation coefficients by Fisher’s z-transformation based on a standard procedure. For this purpose, Spearman’s coefficients were treated as though they were Pearson’s coefficients since the robustness of this assumption is demonstrated in a simulation study (60). Lastly, relationship of the two genetic polymorphisms with PTSD symptom severity was examined in patients using *t*-test. Relations of these polymorphisms with log-transformed cortisol and DHEA-S levels were investigated using a two-way ANOVA with diagnostic groups (patients vs. controls) and genotypes (minor allele carriers vs. non-carriers) as fixed factors.

Additionally, we performed a set of sensitivity analyses to control for the possible confounding effects of age, depression, and anxiety; we first controlled for age and then controlled for age, depression, and anxiety. Specifically, group comparisons were made by an analysis of covariance (ANCOVA) with these variables as covariates. Correlations were examined using unstandardized residuals of the biomarker levels corrected for these variables.

Statistical significance was set at 2-tailed *p* < 0.05 unless otherwise specified. For the correlation between biomarkers and psychological measures, a Bonferroni-corrected *p* < 0.01 (i.e., 0.05/5) was considered as statistically significant, given that five biomarkers were tested. For the SNP analyses, a Bonferroni-corrected *p* < 0.025 (i.e., 0.05/2) was considered as statistically significant, given that two biomarkers (i.e., cortisol and DHEA-S) were tested. All statistical analyses were performed using the Statistical Package for the Social Sciences version 27 (IBM Corp., Tokyo, Japan).

## 3. Results

### 3.1. Demographic, clinical, and psychological characteristics of the sample

Demographic/clinical/psychological variables in patients with PTSD and healthy controls are summarized in Table 1. The participants were all women, and age was well matched between patients and controls. They did not significantly differ in education level, smoking status, or body mass index. Most patients developed PTSD after experiencing interpersonal violence such as physical and/or sexual violence during adulthood, and had been ill for more than 6 months at the time of study participation. Many of them had psychiatric comorbidities, and were receiving psychotropic medications. They were on average moderately to severely ill, as indexed by the mean PDS total score. Compared to controls, patients reported significantly more childhood experiences of maltreatment (assessed with the CTQ), depression and anxiety symptoms (BDI and STAI, respectively), and functional disability (SDISS). For full correlations among all continuous variables in the total sample, see Supplementary Table 1.

**TABLE 1:** Demographic/clinical/psychological characteristics in PTSD patients and healthy controls.

Variable	PTSD patients (*n* = 69)	Healthy controls (*n* = 107)	Analysis		
			**Statistic**	***d.f.***	***P*-value**
Age, years: mean ± SD	37.6 ± 10.0	37.1 ± 13.2	*t* = 0.3	169.7	0.77
Education level^a,b^: median (25th–75th percentile)	3.0 (2.3–4.0)	3.0 (3.0–4.0)	*U* = 3097.5		0.08
Smoking^c^: yes, *n* (%)	8 (11.8)	7 (6.6)	χ^2^ = 1.4	1	0.24
Body mass index^b^: mean ± SD	21.5 ± 3.3	20.9 ± 2.9	*t* = 1.3	173	0.21
Outpatients/inpatients: *n*/*n*	68/1	N.A.			
Type of index trauma^d^: yes, *n* (%)					
Interpersonal violence	60 (88.2)	N.A.			
Accident	4 (5.9)	N.A.			
Others	4 (5.9)	N.A.			
Illness duration, <6 months/ >=6 months^d^: n/n	4/64	N.A.			
Comorbid psychiatric disorder, any: yes, *n* (%)	55 (79.7)	N.A.			
Major depressive disorder	45 (65.2)	N.A.			
Bipolar disorder	5 (7.2)	N.A.			
Anxiety disorder	32 (46.4)	N.A.			
Alcohol/substance abuse or dependence	8 (11.6)	N.A.			
Medication, any^e^: yes, *n* (%)	54 (80.6)	N.A.			
Antipsychotics	18 (26.9)	N.A.			
Antidepressants	39 (58.2)	N.A.			
Anxiolytics	33 (49.3)	N.A.			
Mood stabilizers	8 (11.9)	N.A.			
Hypnotics	27 (40.3)	N.A.			
PDS, total score: mean ± SD	31.3 ± 9.4	N.A.			
Re-experiencing	8.1 ± 3.5	N.A.			
Avoidance	13.8 ± 4.4	N.A.			
Hyperarousal	9.4 ± 3.5	N.A.			
CTQ, total score: mean ± SD	61.0 ± 21.6	36.0 ± 8.8	*t* = 9.1	82.8	**<0.001**
SDISS total score^f^: mean ± SD	19.6 ± 6.3	2.9 ± 4.3	*t* = 19.0	104.1	**<0.001**
BDI-II, total score: mean ± SD	32.2 ± 13.8	5.8 ± 5.1	*t* = 15.3	80.4	**<0.001**
STAI-S score: mean ± SD	51.4 ± 10.1	36.7 ± 7.9	*t* = 10.3	119.7	**<0.001**
STAI-T score: mean ± SD	63.0 ± 8.4	39.1 ± 8.9	*t* = 17.7	174	**<0.001**

PTSD, posttraumatic stress disorder; PDS, posttraumatic diasnostic scale; CTQ, childhood trauma questionnaire; SDISS, Sheehan Disability Scale; BDI-II, beck depression inventory-II; STAI-S, state-trait anxiety inventory-state; STAI-T, state-trait anxiety inventory-trait; d.f., degree of freedom; SD, standard deviation; N.A., not applicable.

Bold values indicate significant results.

^a^Coded as follows: 1, junior high school graduate; 2, high school graduate; 3, some college graduate/partial university; 4, university graduate; 5, graduate school graduate.

^b^*n* = 68 for patients and *n* = 107 for controls.

^c^*n* = 68 for patients and *n* = 106 for controls.

^d^*n* = 68 for patients.

^e^*n* = 67 for patients.

^f^*n* = 67 for patients and *n* = 107 for controls.

### 3.2. Potentially confounding variables

Age was significantly correlated negatively with ACTH, DHEA-S, and renin levels in patients, and significantly correlated positively with cortisol and negatively with DHEA-S, renin, and aldosterone levels in controls (all *p* < 0.05). Age was not correlated with PTSD severity (including the PDS total score and three subscale scores) in patients or with childhood maltreatment history (CTQ total score) in patients or in controls (all *p* > 0.8). Levels of the five hormones did not significantly differ between patients treated with and those without any class of medication, including antipsychotics, antidepressants, anxiolytics, mood stabilizers, and hypnotics (all *p* > 0.05), except for significantly higher cortisol levels in patients treated with mood stabilizers than those without (*U* = 383.5, *p* = 0.004). The five hormonal levels did not significantly differ between patients with comorbid major depressive disorder and those without (all *p* > 0.05). For menstrual cycles, the numbers of participants who endorsed “before menstruation (within 1 week),” “during menstruation,” “after menstruation (within 1 week),” and “after menopause” did not significantly differ between patients and controls (χ^2^ = 6.5, *p* = 0.09). In addition, five participants (all patients) were taking oral contraceptives. Patients taking oral contraceptives showed significantly higher cortisol levels than those without (*U* = 259.0, *p* = 0.014), while no significant differences were seen in the other four hormone levels (all *p* > 0.1). We therefore excluded cortisol data for these five patients in subsequent analyses; these five patients did not differ from the other 64 patients in any of the psychological characteristics including CTQ total, PDS total and three subscales, SDISS total, BDI-II total, and STAI-S/-T (all *p* > 0.2 by Mann–Whitney *U*-test). Levels of cortisol, ACTH, DHEA-S, renin, and aldosterone were not significantly different between the three study sites (all *p* > 0.05 by the Kruskal–Wallis test).

### 3.3. Biomarkers in patients vs. controls and their relation with clinical/psychological characteristics

As shown in Table 2, no significant differences were seen between patients and controls in any of the five markers, including cortisol, ACTH, DHEA-S, renin, and aldosterone.

**TABLE 2:** Blood levels of HPA axis and RAA system hormones in patients and controls.

	PTSD patients (*n* = 69)	Healthy controls (*n* = 107)	Mann–Whitney *U*-test	
			***U***	***P***
Cortisol (μg/dl)^a^	7.0 (5.5–10.5)	7.3 (5.6–9.3)	3587.5	0.60
ACTH (pg/ml)	14.5 (10.3–19.9)	16.7 (10.5–21.7)	3309.0	0.25
DHEA-S (μg/dl)	138.0 (100.5–210.0)	178.0 (108.0–239.0)	3287.0	0.22
Renin (pg/ml)^b^	8.3 (4.1–15.2)	7.8 (4.4–12.5)	3144.5	0.44
Aldosterone (pg/ml)^b^	93.3 (51.9–144.5)	80.0 (52.1–143.0)	3036.0	0.70

PTSD, posttraumatic stress disorder; ACTH, adrenocorticotropic hormone; DHEA-S, dehydroepiandrosterone-sulfate. Data are shown as “median (25th–75th percentile).”

^a^*n* = 64 for patients and *n* = 107 for controls.

^b^*n* = 61 for patients and *n* = 96 for controls.

**FIGURE 1:**
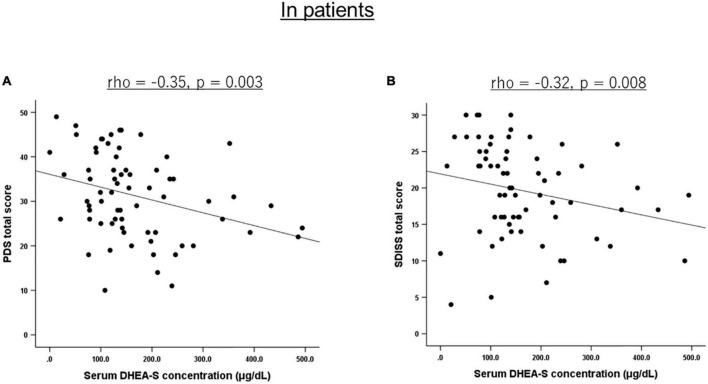
Scatterplot showing the relationship of serum DHEA-S levels with PTSD severity and functional disability in patients. In PTSD patients, serum DHEA-S levels were significantly negatively correlated with **(A)** overall PTSD severity assessed with the PDS total score and **(B)** functional disability assessed with the SDISS total score. Correlation was calculated by the Spearman’s rank order correlation.

Correlations of the five biomarkers with PTSD symptomatology (PDS) and functional disability (SDISS) in patients are presented in Table 3. DHEA-S levels were significantly negatively correlated with overall PTSD severity (rho = −0.350, *p* = 0.003; Figure 1A), hyperarousal symptom (rho = −0.386, *p* = 0.001), and functional disability (rho = −0.322, *p* = 0.008; Figure 1B). Comparisons of correlations in patients vs. controls revealed that the association between lower DHEA-S levels and greater functional disability was significantly different between the groups, indicating that this association was specific to patients (Table 4). Cortisol, ACTH, renin, and aldosterone levels were not significantly correlated with any symptoms or functional disability in patients or in controls (Tables 3, 4).

**TABLE 3:** Correlations of psychological characteristics with HPA axis and RAA system hormones in PTSD patients.

	Cortisol^a^	ACTH^b^	DHEA-S^b^	Renin^c^	Aldosterone^c^
PDS total score	−0.108	−0.210	−0.350**	−0.012	0.129
PDS Re-experiencing	−0.055	−0.186	−0.236	−0.153	0.044
PDS Avoidance	−0.132	−0.157	−0.266	0.070	0.103
PDS Hyperarousal	−0.061	−0.186	−0.386**	0.062	0.110
CTQ total score	−0.071	0.188	−0.042	−0.019	−0.072
SDISS total score	−0.297	−0.245	−0.322**	0.033	0.077
BDI-II total score	−0.179	−0.078	−0.236	0.140	0.214
STAI-S score	−0.101	−0.013	0.025	0.023	−0.042
STAI-T score	−0.086	−0.005	−0.183	0.090	0.018

PTSD, posttraumatic stress disorder; ACTH, adrenocorticotropic hormone; DHEA-S, dehydroepiandrosterone-sulfate; PDS, posttraumatic diagnostic scale; CTQ, childhood trauma questionnaire; SDISS, Sheehan Disability Scale; BDI-II, beck depression inventory-II; STAI-S, state-trait anxiety inventory-state; STAI-T, state-trait anxiety inventory-trait.

**p < 0.01 (significant). Correlations were calculated using Spearman’s rho.

^a^*n* = 64 for PDS, CTQ, BDI-II, and STAI, and *n* = 63 for SDISS.

^b^*n* = 69 for PDS, CTQ, BDI-II, and STAI, and *n* = 67 for SDISS.

^c^*n* = 61 for PDS, CTQ, BDI-II, and STAI, and *n* = 59 for SDISS.

**TABLE 4:** Correlations of psychological characteristics with HPA axis and RAA system hormones in healthy controls (including differences in correlations between patients and controls).

	Cortisol^a^	ACTH^a^	DHEA-S^a^	Renin^b^	Aldosterone^b^
CTQ total score	−0.161	−0.041	0.001	0.017	0.233
Difference vs. patients	*Z* = 0.57	*Z* = 1.47	*Z* = 0.27	*Z* = 0.22	*Z* = 1.85
SDISS total score	−0.118	−0.088	0.152	−0.044	0.164
Difference vs. patients	*Z* = 1.16	*Z* = 1.02	*Z* = 3.07**	*Z* = 0.46	*Z* = 0.52
BDI-II total score	−0.009	0.047	−0.001	−0.012	0.106
Difference vs. patients	*Z* = 1.07	*Z* = 0.80	*Z* = 1.52	*Z* = 0.91	*Z* = 0.66
STAI-S score	0.115	0.057	−0.103	0.004	0.114
Difference vs. patients	*Z* = 1.34	*Z* = 0.45	*Z* = 0.82	*Z* = 0.11	*Z* = 0.94
STAI-T score	0.119	0.097	−0.075	−0.048	0.060
Difference vs. patients	*Z* = 1.28	*Z* = 0.65	*Z* = 0.70	*Z* = 0.82	*Z* = 0.25

ACTH, adrenocorticotropic hormone; DHEA-S, dehydroepiandrosterone-sulfate; CTQ, childhood trauma questionnaire; SDISS, Sheehan Disability Scale; BDI-II, beck depression inventory-II; STAI-S, state-trait anxiety inventory-state; STAI-T, state-trait anxiety inventory-trait.

**p < 0.01 (significant). Correlations were calculated using Spearman’s rho. Differences in correlations between patients and controls were determined by Fisher’s Z transformation.

^a^*n* = 107.

^b^*n* = 96.

Correlations between the CTQ total score and five biomarkers are shown in Table 3 for patients and Table 4 for controls. Childhood maltreatment history (CTQ total score) was not significantly correlated with any of the five biomarkers in patients or in controls. Considering that patients and controls did not differ in the five biomarker levels, we additionally examined the correlations between the CTQ total score and these markers in the total sample (i.e., patients and controls were combined) and found no significant correlation for any of the five markers (all *p* > 0.1; Supplementary Table 1).

Depression (BDI-II) and anxiety (STAI-S/-T) were not significantly correlated with any of the five biomarkers either in patients (Table 3) or in controls (Table 4).

### 3.4. Effects of genetic variations on biomarkers

Numbers of participants with the *FKBP5* rs1360780 CC, CT, and TT genotypes were 28 (54.9%), 19 (37.3%), and 4 (7.8%) in patients (respectively) and 57 (60.6%), 30 (31.9%), and 7 (7.4%) in controls (respectively). Those with the *CACNA1C* rs1006737 GG, GA, and AA genotypes were 44 (86.3%), 7 (13.7%), and 0 (0.0%) in patients (respectively) and 77 (81.9%), 17 (18.1%), and 0 (0.0%) in controls (respectively). For both SNPs, genotype frequencies did not deviate from Hardy-Weinberg equilibrium either in patients or in controls (all *p* > 0.1). Due to the small number of the rs1360780 TT genotype, we combined the TT genotype with the CT genotype in all analyses, as in our previous study (61); thus, all comparisons were made with the two groups, i.e., CC vs. CT + TT genotypes. Likewise, as there were no participants with the rs1006737 AA genotype, all comparisons were made with the remaining two groups, i.e., GG vs. GA genotypes.

PTSD severity as assessed with the PDS total score in patients was not significantly different between the *FKBP5* rs1360780 genotype groups (*t* = 0.56, *p* = 0.58) or between the *CACNA1C* rs1006737 genotype groups (*t* = 0.34, *p* = 0.74).

For the *FKBP5* polymorphism, the two-way ANOVA for log-transformed cortisol levels showed no significant main effect of diagnostic group (*p* = 0.25) or rs1360780 genotype (*p* = 0.13) or their interaction (*p* = 0.26). Similarly, the two-way ANOVA for log-transformed DHEA-S levels showed no significant main effect of diagnostic group (*p* = 0.52) or rs1360780 genotype (*p* = 0.81) or their interaction (*p* = 0.23).

**FIGURE 2:**
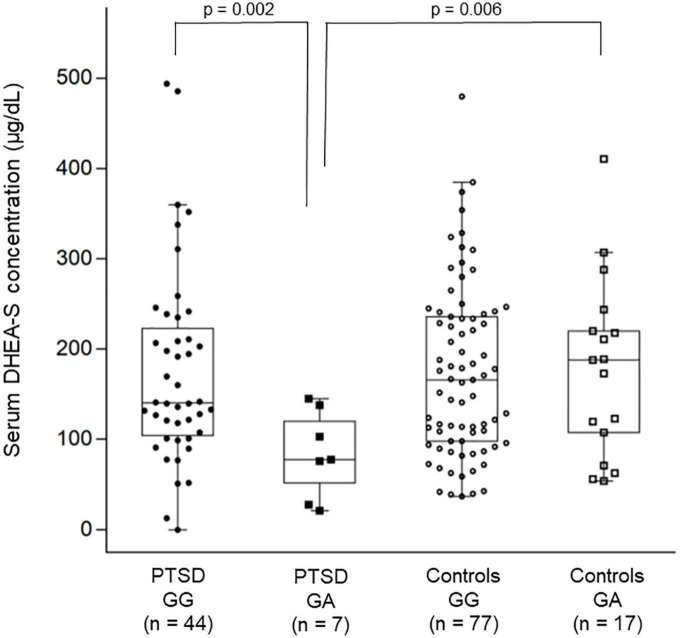
Combined dot- and box-plot showing the comparison of serum DHEA-S levels as a function of diagnostic groups and the *CACNA1C* rs1006737 genotype groups. Comparison of log-transformed DHEA-S levels was made by the two-way ANOVA. In this figure, raw data of DHEA-S levels without log-transformation is presented.

For the *CACNA1C* polymorphism, the two-way ANOVA for log-transformed cortisol levels showed no significant main effect of diagnostic group (*p* = 0.42) or rs1006737 genotype (*p* = 0.51) or their interaction (*p* = 0.93). However, the two-way ANOVA for log-transformed DHEA-S levels showed a significant main effect of diagnostic group (*p* = 0.013), rs1006737 genotype (*p* = 0.014), and their interaction (*p* = 0.009); this analysis further revealed that patients with the GG genotype and those with the GA genotype significantly differed in DHEA-S levels (*p* = 0.002) while controls with the GG genotype and those with the GA genotype did not differ in DHEA-S levels (*p* = 0.89) and that patients with the GG genotype and controls with the GG genotype did not differ in DHEA-S levels (*p* = 0.87) while patients with the GA genotype and controls with the GA genotype significantly differed in DHEA-S levels (*p* = 0.006); patients with the GA genotype had lower DHEA-S levels than patients with the GG genotype and controls with the GA genotype (Figure 2). To confirm this result using the raw DHEA-S data (i.e., without log-transformation), we further used the Mann–Whitney *U*-test to compare DHEA-S levels between patients with the GG genotype and those with the GA genotype and between patients with the GA genotype and controls with the GA genotype, which yielded similar results (*p* = 0.017 and *p* = 0.034, respectively).

### 3.5. Sensitivity analysis to control for possible confounding effects of age, depression, and anxiety

An ANCOVA was used to compare log-transformed levels of the five hormones between patients and controls with age as a covariate, which confirmed no significant difference between groups in any of the five hormones (all *p* > 0.3), as in the original analysis. An additional age-, depression- (BDI-II), and anxiety- (STAI-S and –T) corrected ANCOVA also confirmed no significant difference between groups in any of the five hormones (all *p* > 0.2).

We also examined whether the main significant findings were robust to the effect of potential confounders. Specifically, Spearman’s correlations between the PDS/SDISS scores and DHEA-S levels in patients were calculated using unstandardized residuals of the DHEA-S levels corrected for age. This analysis confirmed significant correlations for all the PDS/SDISS indices, including PDS total (rho = −0.435, *p* < 0.001), re-experiencing (rho = −0.273, *p* = 0.023), avoidance (rho = −0.333, *p* = 0.005), hyperarousal (rho = −0.502, *p* < 0.001), and SDISS total (rho = −0.352, *p* = 0.003). An additional correlation analysis between the PDS/SDISS scores and DHEA-S levels in patients corrected for age, depression (BDI-II), and anxiety (STAI-S and –T) confirmed similar results for PDS total (rho = −0.366, *p* = 0.002), re-experiencing (rho = −0.250, *p* = 0.038), avoidance (rho = −0.257, *p* = 0.033), hyperarousal (rho = −0.434, *p* < 0.001), and SDISS total (rho = −0.259, *p* = 0.035).

In addition, we performed an age-corrected two-way (i.e., diagnostic group*rs1006737 genotype) ANCOVA to compare log-transformed DHEA-S levels, and confirmed the significant main effect of genotype (*p* = 0.020), while main effect of group (*p* = 0.077) and interaction (*p* = 0.11) were not significant. An additional age-, depression- (BDI-II), and anxiety- (STAI-S and –T) corrected two-way ANCOVA for log-transformed DHEA-S levels also confirmed the significant main effect of genotype (*p* = 0.014), but not main effect of group (*p* = 0.79) or interaction (*p* = 0.076).

### 3.6. DEX-induced HPA axis negative feedback in an independent sample

We performed the DEX suppression test in the independent sample of 33 healthy women. Median (25th–75th percentile) levels of basal and post-DEX cortisol (μg/dl) were 9.7 (8.1–12.5) and 1.2 (0.0–1.9), respectively; their correlation failed to reach statistical significance (rho = 0.34, *p* = 0.054). Basal and post-DEX DHEA-S levels (μg/dl) were 128.0 (95.5–171.5) and 92.0 (66.5–117.0), respectively; their correlation was highly significant (rho = 0.94, *p* < 0.001). These results indicated that post-DEX cortisol levels could be only weakly predicted by its basal levels, whereas post-DEX DHEA-S levels could be almost totally predicted by basal levels.

## 4. Discussion

HPA axis in individuals with trauma and PTSD has been extensively investigated for more than three decades, and several important aspects of stress hormone dysregulation associated with these conditions have been identified. However, a substantial proportion of studies have not replicated these findings, and the controversy also exists even in meta-analytic studies (3–5). To overcome this, studies have used various approaches, including different paradigms of hormone measurement (e.g., baseline, pharmacological, and psychosocial challenge), different samples (e.g., blood, saliva, urine, and hair), and different participant characteristics (e.g., controls with and without trauma). Still, the nature of HPA axis alterations in PTSD and trauma remains somewhat elusive.

The present study attempted to better understand the HPA axis (dys) function in PTSD by simultaneously examining RAA system and genetic polymorphisms of candidate genes. The main finding of this study was that DHEA-S levels were significantly negatively correlated with PTSD severity and functional disability. Furthermore, patients with the *CACNA1C* rs1006737 GA genotype had significantly lower DHEA-S levels than patients with the GG genotype and controls with the GA genotype. Childhood maltreatment history was not significantly associated with the five hormonal levels.

None of the HPA axis hormones measured were significantly different between PTSD patients and healthy controls. This negative result is not surprising given the mixed findings of previous studies on HPA axis in PTSD, some of which did not find significant case-control difference like the present study [e.g., (5)]. Nonetheless, DHEA-S levels were significantly negatively correlated with PTSD severity and functional disability. The comparison of correlations in patients vs. controls revealed that this association between lower DHEA-S levels and greater functional disability was specific to patients. This suggests that DHEA-S may have protective effects in these individuals, being in line with previous findings (11, 14). Consistently, a recent study reported significant negative association between blood DHEA-S levels and PTSD severity *via* personality traits (62). Our finding may also be related to the fact that circulating DHEA-S is relatively stable and that the basal DHEA-S level corresponds well to negative feedback inhibition to the DEX suppression test, given that such a biomarker that is relatively robust to intra- and inter-daily variations and measurement paradigms, compared to the one strongly affected by these factors, can better reflect psychopathology.

HPA axis is involved in stress response in concert with the immune/inflammatory system and autonomic nervous system, and indeed, these systems have also been implicated in the pathophysiology of PTSD (2, 63). Moreover, DHEA(S) has been shown to attenuate inflammatory responses (64, 65). It is therefore possible that alterations in DHEA(S) and HPA axis function may be involved in the biological mechanism of PTSD by interacting with the immune and inflammatory system. From a clinical viewpoint, there are currently no established biomarkers for the diagnosis or prognosis of PTSD. In this regard, our finding suggests that DHEA-S may serve as a marker for severity and disability of PTSD, although this possibility needs to be further investigated in future studies.

Renin and aldosterone levels did not significantly differ between patients and controls or correlate with childhood maltreatment history. On the other hand, a preceding study reported that plasma levels of renin, but not aldosterone, were increased in persons with PTSD than controls without trauma (27). In addition, another study found that childhood maltreatment was associated with increased aldosterone but not renin levels (28). This inconsistency may be due to the relatively small sample in the present study. It may also be possible that medications for our patients have normalized these hormone levels. As there have been only a few studies investigating the RAA system in relation to PTSD or childhood trauma, more research is needed to understand the role of this system in these conditions.

*CACNA1C* rs1006737 genotype was significantly associated with DHEA-S levels in patients, suggesting that the observed relation between DHEA-S and PTSD symptomatology could be at least in part influenced by this SNP. This observation is in accordance with a previous finding that this SNP can interact with early life stress to affect HPA axis reactivity (35). Rodent studies have shown that chronic stress and glucocorticoids can affect Cav1.2 mRNA expression encoded by *Cacna1c* (66–68). We found that minor A-allele carriers had lower DHEA-S levels than G-allele homozygotes. This pattern of unfavorable phenotypes related to the A-allele compared to G-allele accords with previous findings obtained in various populations (35, 39). In addition, the A-allele is shown to be associated with decreased *CACNA1C* expression (40, 41). On the other hand, the *CACNA1C* rs1006737 was not significantly associated with PTSD symptomatology. Moreover, the *FKBP5* rs1360780 was not significantly associated with any hormonal levels or symptomatology. It should be noted here that there are considerable ethnic differences in minor allele frequencies of these SNPs. The minor T-allele frequency of *FKBP5* rs1360780 and the minor A-allele frequency of *CACNA1C* rs1006737 in our sample were 0.245 and 0.083 (respectively), being consistent with the frequency of 0.227 and 0.059 (respectively) reported in a representative genome variation database of Japanese individuals (69). In contrast, the frequency of rs1360780 T-allele and that of rs1006737 A-allele are reported to be around 0.30 and 0.35 (respectively) among many other populations such as Europeans, according to the Genome Aggregation Database (gnomAD). Thus, our non-significant results may be attributable to the lower minor allele frequencies of these SNPs in Japanese, as well as the small sample size.

Our findings that the *CACNA1C* rs1006737 risk A-allele is associated with lower DHEA-S levels and that lower DHEA-S levels are related to more severe PTSD collectively suggest that genetic risk factors affect the stress response system, namely HPA axis, and as a result influence the disorder. In line with this possibility, it is widely accepted that both genetic factors and environmental stimuli, and the resultant gene-environment interaction, are involved in the etiology of PTSD (70). The present findings also suggest that rs1006737 and DHEA-S levels contribute to subtypes (or outcomes) within PTSD rather than the development of this disorder, considering that DHEA-S levels did not differ between patients and controls. Supporting this, it is postulated that PTSD is a heterogeneous disorder with distinct subtypes (71). While our findings suggest a role of *CACNA1C* and HPA axis, further studies are necessary to get a bigger picture of the gene-environment interaction in PTSD and subtypes of this disorder.

This study has several limitations. First, the sample size was not very large, particularly for the genetic analyses. For example, recent genome-wide association studies of PTSD and trauma have employed tens of thousands of participants (72, 73). It is therefore possible that some of our non-significant results might have been type II errors. It may be worth noting, however, that the present study adopted a hypothesis-based, candidate gene approach focusing on symptoms and biomarkers rather than case-control comparison. As a result, we observed a significant association between the *CACNA1C* risk allele and DHEA-S levels. Second, this cross-sectional study did not provide information as to the causality between trauma/PTSD and hormonal alterations. Future longitudinal studies are needed to draw any conclusion as to the causal relationship. Third, the hormonal measurement was conducted only once without any stimulations, although our additional DEX suppression test indicated that DEX-stimulated DHEA-S levels showed very high correlation with its basal levels. Still, given that HPA axis hormones, particularly cortisol, exhibit significant diurnal variations, it would be more informative to collect samples at multiple time points. In addition, caution should be exercised when extrapolating the DEX suppression test results obtained in the healthy sample to the PTSD sample. Fourth, this study included only female participants, which may have affected the findings. For instance, HPA axis function is shown to be different between men and women (74) and the heritability of PTSD is suggested to be different between sexes (75). Thus, our findings might represent some unique features of women with PTSD. Fifth, data of several participants were unavailable or excluded for some biomarkers and SNPs, which might have affected the results. Sixth, while the amount of liquid consumption can potentially influence the RAA system, such data were not collected in this study. Seventh, we used the DSM-IV-based scale (i.e., PDS), but not an updated DSM-5-based one, for the diagnosis of PTSD. While this was because we were not aware of any Japanese version of DSM-5-based scale for the diagnosis of PTSD at the time of the study initiation, differences in diagnostic criteria such as the absence and presence of the “negative alterations in cognitions and mood” criterion between DSM-IV and DSM-5 (respectively) might have affected the results. Finally, since most of our patients were receiving psychotropics, the possibility cannot be ruled out that such medication may have influenced the HPA axis and/or RAA system, even though we did not observe any significant relationship between medication and hormonal levels except for the relation between the use of mood stabilizers and cortisol levels.

In summary, this study shows that lower DHEA-S levels could indicate more severe subtype of PTSD, the association of which might be partly modified by the *CACNA1C* genetic polymorphism. Our findings suggest an involvement of DHEA-S in the pathophysiology of PTSD and further point to the importance of considering genetic factors in investigating stress response alterations in trauma and PTSD. Since neuroendocrine responses to stress depend on developmental timing, duration, time of day, and nature of stressors (76), future studies that address these issues would be necessary to get the bigger picture of HPA axis dysfunction in trauma-related conditions.

## Data availability statement

The datasets presented in this study can be found in online repositories. The names of the repository/repositories and accession number(s) can be found below: https://www.ncbi.nlm.nih.gov/clinvar/, SCV002587063 (for rs1360780) and SCV002588462 (for rs1006737).

## Ethics statement

The studies involving human participants were reviewed and approved by the Ethics Committee of National Center of Neurology and Psychiatry, Ethics Committee of Tokyo Women’s Medical University, and Ethics committee of Nagoya City University. The patients/participants provided their written informed consent to participate in this study.

## Author contributions

RK and HH designed the study, undertook the statistical analyses, and wrote the draft of the manuscript. RK, HH, MI, ML, MaN, MeN, KI, RI, DS, and YK collected the data. FY performed the genotyping. DS, TK, HK, and YK gave critical comments on the manuscript. All authors contributed to the article and approved the final manuscript.
